# The standard of healthcare accreditation standards: a review of empirical research underpinning their development and impact

**DOI:** 10.1186/1472-6963-12-329

**Published:** 2012-09-20

**Authors:** David Greenfield, Marjorie Pawsey, Reece Hinchcliff, Max Moldovan, Jeffrey Braithwaite

**Affiliations:** 1 Centre for Clinical Governance Research, Australian Institute of Health Innovation, University of New South Wales, Sydney, New South Wales, 2052, Australia

**Keywords:** Healthcare, Accreditation, Standards, Evidence for use, Narrative literature review

## Abstract

**Background:**

Healthcare accreditation standards are advocated as an important means of improving clinical practice and organisational performance. Standard development agencies have documented methodologies to promote open, transparent, inclusive development processes where standards are developed by members. They assert that their methodologies are effective and efficient at producing standards appropriate for the health industry. However, the evidence to support these claims requires scrutiny. The study’s purpose was to examine the empirical research that grounds the development methods and application of healthcare accreditation standards.

**Methods:**

A multi-method strategy was employed over the period March 2010 to August 2011. Five academic health research databases (Medline, Psych INFO, Embase, Social work abstracts, and CINAHL) were interrogated, the websites of 36 agencies associated with the study topic were investigated, and a snowball search was undertaken. Search criteria included accreditation research studies, in English, addressing standards and their impact. Searching in stage 1 initially selected 9386 abstracts. In stage 2, this selection was refined against the inclusion criteria; empirical studies (n = 2111) were identified and refined to a selection of 140 papers with the exclusion of clinical or biomedical and commentary pieces. These were independently reviewed by two researchers and reduced to 13 articles that met the study criteria.

**Results:**

The 13 articles were analysed according to four categories: overall findings; standards development; implementation issues; and impact of standards. Studies have only occurred in the acute care setting, predominately in 2003 (n = 5) and 2009 (n = 4), and in the United States (n = 8). A multidisciplinary focus (n = 9) and mixed method approach (n = 11) are common characteristics. Three interventional studies were identified, with the remaining 10 studies having research designs to investigate clinical or organisational impacts. No study directly examined standards development or other issues associated with their progression. Only one study noted implementation issues, identifying several enablers and barriers. Standards were reported to improve organisational efficiency and staff circumstances. However, the impact on clinical quality was mixed, with both improvements and a lack of measurable effects recorded.

**Conclusion:**

Standards are ubiquitous within healthcare and are generally considered to be an important means by which to improve clinical practice and organisational performance. However, there is a lack of robust empirical evidence examining the development, writing, implementation and impacts of healthcare accreditation standards.

## Background

In health accreditation a standard is “a desired and achievable level of performance against which actual performance is measured” [1]. Standards enable “health service organisations, large and small, to embed practical and effective quality improvement and patient safety initiatives into their daily operations” [2]. External organisational and clinical accreditation standards are considered necessary to promote high quality, reliable and safe products and services [2,3]. There are over 70 national healthcare accreditation agencies worldwide that develop or apply standards, or both, specifically for health services and organisations [4].

The International Society for Quality in Health Care (ISQua) seeks to guide and standardise the development of these agencies and the standards they implement [5]. ISQua advocates that accreditation standards themselves need to meet exacting standards, and has standards for how to develop, write and apply them. ISQua conducts the International Accreditation Program (IAP) for the certification or accreditation of standards against their standards [5]. The International Standards Organisation (ISO), a network of the national standards institutes of 162 countries, is the largest developer and publisher of international standards [6]. Standards from ISO are also applied in international health jurisdictions.

In short, healthcare standards, and standards for standards, are ubiquitous. They are advocated to be an important means of improving clinical practice and organisational performance. ISQua, and many national bodies, espouse, and have documented methodologies to promote open, transparent, inclusive development processes where standards are developed by members [6-11]. They assert that their methodologies are effective and efficient at producing standards appropriate for the health industry. However, the evidence to support these claims requires scrutiny. What is the basis to ground the standard development methodologies in use? What research demonstrates how standards should be crafted and structured to ensure they are understandable, unambiguous, achievable and reliable in making assessments? What studies have identified the necessary steps to enable standards to be incorporated into everyday practice? Is there evidence to show whether standards improve practice? The purpose of this study was to examine these questions by identifying and analysing the research literature focusing on the development methods and application of healthcare accreditation standards.

The analysis is a systematic narrative synthesis of the literature [12]. The intention is to generate new insights and bring transparency to the topic under investigation [13,14]. This type of review is appropriate for this topic for four reasons. First, the review aims to examine a complex initiative applied in diverse contexts [15]. That is, accreditation programs are complex organisational interventions, trying to shape both organisational and clinical conduct, within a multifaceted context in turn shaped by, for example, the healthcare and policy environment. Second, accreditation programs, involving healthcare standards, have been researched in different ways by divergent groups. The analysis method adopted here is intended specifically for interventions researched in a myriad of ways [12]. Third, the approach enables consideration of apparently disparate data generated by research into accreditation standards, as a complex organisational intervention [15]. Fourth, the questions being investigated are preliminary questions that need to be asked of this intervention and the approach is designed exactly for this [14,15]. The review differs from previous reviews [16,17] in being specifically focused only on healthcare accreditation standards and not the broader “standards” field. This review is the first to undertake a systematic and detailed narrative synthesis of accreditation standards.

## Methods

### Selection criteria and search strategy

The selection criteria were: peer-reviewed, publicly available English language empirical research papers on the topic of healthcare accreditation standards. Discussion and commentary, and non-English language papers were excluded. Despite these focused criteria, we recognise that they may capture heterogeneous literature including, possibly, an overlap with work covering other forms of regulation. To counter this potential problem we used a staged search strategy to identify and remove any papers not focused on the study topic. This approach is valid for two reasons. First, there are overlaps between how regulatory strategies are at times discussed in the literature [18-20]. The reviewing of abstracts or the full papers provided a mechanism by which to screen out literature not on the study topic. Second, previous reviews and a preliminary investigation signalled that empirical research literature available on standards was limited.

**Figure 1:**
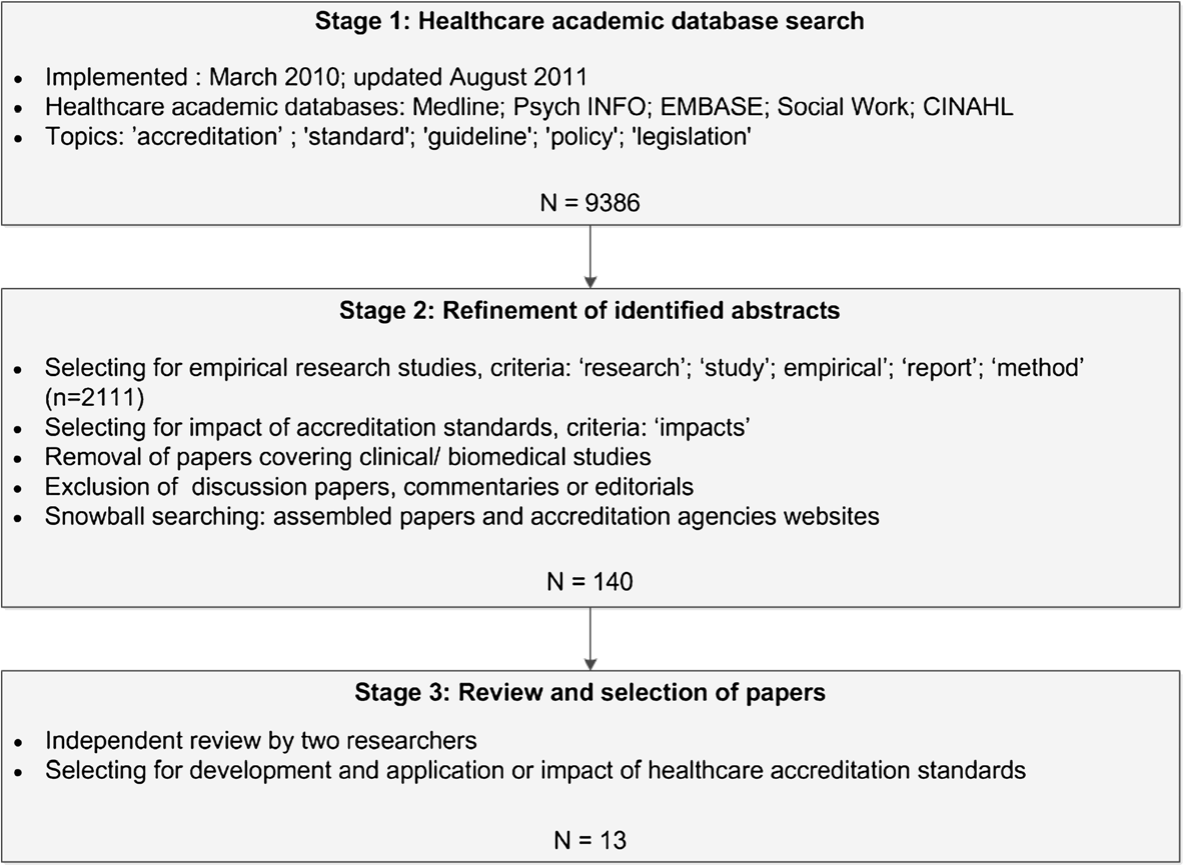
Literature search, review and selection flow chart.

A multi-method strategy based on similar review designs was employed [16,21,22]. There were three stages (see Figure 1). The search was first conducted in March 2010 and updated in August 2011. Citations and abstracts that met the search criteria were downloaded into Endnote X.0.2, a reference management program. Abstracts and, where uncertainty arose, complete papers, were reviewed against the selection criteria for inclusion in the review. 

The first stage had three steps. First, we selected databases in the health sector. Literature was drawn from five electronic bibliographic databases: Medline, Psych INFO, EMBASE and Social Work databases from 1980, and CINAHL (nursing and allied health literature) from 1982. Second, we identified abstracts focusing on the topic of ‘accreditation’. Third, we selected abstracts using the terms ‘standard’, ‘guideline’, ‘policy’ and ‘legislation’; where appropriate, terms were truncated with the symbol ‘$’ and searched using the ‘Exp’ function to capture widest publication of papers (for example, guideline$ or polic$). The initial search yielded 9386 abstracts (including duplicates). We reviewed the selection to exclude those not written in English and also to remove duplicates.

In the second stage we refined the collected abstracts. Two researchers independently reviewed the abstracts, selecting papers using two criteria. We selected for empirical research studies, using derivations of phrases such as ‘research’, ‘study’, ‘empirical’ or ‘report’, and ‘method’. Using this strategy the selection was reduced to 2111 articles. This group was further analysed to identify those papers that covered ‘impacts’ of accreditation standards. At this point we removed papers covering clinical or biomedical issues and also discussion pieces, commentaries or editorials. To supplement the formal search process, two less structured search methods were implemented. We undertook a 'snowballing' search, which is a variation on snowballing sampling [23]. That is, we examined the assembled manuscripts reference lists for additional relevant papers potentially missed in the formal search. In parallel, an investigation of websites of agencies associated with the study topic, that is, reports or papers investigating the evidence base for accreditation or quality standards in the health sector, was conducted. We searched: the ISQua research site; the websites of 31 healthcare accreditation agencies worldwide; ISO website; and standards organisations’ websites of a number of countries (Additional file 1: Appendix 1). The application of the stage 2 refinement processes to the collected abstracts yielded 140 articles.

In the third stage, to determine the final selection of papers meeting the study criteria, two experienced researchers independently reviewed the identified 140 papers and discussed their relevance. The focus was the selection of papers that addressed development methods and application of healthcare accreditation standards. This stage derived 13 articles.

### Analysis

The selected papers were analysed by three independent researchers in two ways. First, the characteristics of the studies were noted. For each paper a summary of authors, country, sector, aim, methods, major findings and conclusions, and study quality was compiled. The level of evidence was assessed using Australian National Health and Medical Research Council guidelines [24] and study quality by an assessment tool developed from publically available checklists [21,25]. Together they enabled examination of study quality, incorporating intervention or aetiology (that is, impact), level of evidence, design and appraisal of quality (Table 1). Second, a narrative analysis of the literature was conducted in line with the study aims. 

**Table 1:** Quality rating assessment criteria*

**For all study designs**	**Assessment criteria**
	Clearly specified and appropriate research question
	Clear details and justification of study design, including selection of cases and controls
	Detailed description of research setting, data collection methods and type of analysis performed
	Logical presentation and discussion of results and study conclusions
	Adequate sample size and response rate (>60%) relative to study design
**Overall ratings**	**Assessment criteria**
+++	All of the above criteria fulfilled
++	Almost all of the above criteria fulfilled, and those criteria that were not fulfilled were thought unlikely to alter the conclusions of the study
+	Some of the criteria were fulfilled, and those criteria that were not fulfilled were thought unlikely to alter the conclusions of the study

*adapted from Cunningham et al. 2011.

## Results

The 13 papers were synthesised (Table 2). The results are presented under three headings: standards development; implementation issues; and the impact of standards. The papers were examined according to date of publication, country, sector, methodology and focus.

**Table 2:** Assessment of empirical healthcare standards research

**Study details**			**Study characteristics**			**Study quality**			
**Author-year**	**Country**	**Sector**	**Aim**	**Methods**	**Major findings and conclusions**	**Intervention or Aetiology (I or A) (NHMRC hierarchy)**	**Level of evidence (NHMRC hierarchy)**	**Design**	**Quality rating**
Aiken et al. (2008) [35]	United Kingdom	Acute care	To test the impact of the implementation of Magnet principles of improving nurses’ work environments.	Survey Comparison with national sample	Pre-survey: nurse work environment was less positive and they experienced less job satisfaction than the national sample.	I	IV	Pre- and post- evaluation	+++
								Compar-ative study	
					Post-survey: significant improvement in nurse practice environment, job satisfaction and appraisals of the quality of patient care; practice environment better than national sample. The implementation of the Magnet hospital program was associated with a significantly improved nursing work environment as well as improved job-related outcomes for nurses and markers for quality of patient care.				
Devers, Pham, Liu (2004) [34]	United States	Acute care	To describe hospitals’ patient-safety initiatives, and the relative roles that regulation, markets and professionalism have in stimulating progress.	Interviews with stakeholders	Hospitals’ major patient-safety initiatives were primarily intended to meet Joint Commission (JC) requirements.	A	IV	Cross sectional	+++
				Database analysis (CTS patient safety and Leapfrog Group survey data)	Internal (professionalism, resources), external (regulation, markets) and contextual (research, organisational factors) facilitators and barriers identified.				
					Impact on hospitals of increased attention to patient safety has been mixed and on patients it is unclear, because relevant data did not exist or were difficult to interpret. Professional and market initiatives have facilitated improvement, however quasi-regulatory forces, such as JC, are having the greatest impact on hospitals’ patient-safety efforts.				
Herr, Titler (2009) [17]	United States	Acute care	To examine compliance with the new pain assessment and management standards of the Joint Commission on Accreditation of Healthcare Organizations (JCAHO) for accredited health care organisations.	Archival documents analysis	Trends over time illustrate improvements in pain assessment practices, with a majority of patients having some documentation related to pain. However, only just over half the patients had medicines ordered. Practice improvement in the administration of medicine was noted.	A	III-2	Retrospective cross sectional, cohort study	+++
					Pain assessment and management practices in the emergency departments showed improvements in line with the introduction of standards.				
Kozhimannil et al. (2009) [30]	Philippines	Acute care	To examine the population-level impacts of two programs – national health insurance (PhilHealth) and a donor funded franchise of midwife clinics (Well Family Midwife Clinics) on achievement of minimum standards for prenatal and delivery care.	Survey	The PhilHealth insurance program scale was associated with increased odds of receiving at least four prenatal visits and receiving a visit during the first trimester of pregnancy. Exposure to midwife clinics was not associated with significant changes in achievement of prenatal care standards. The expansion of an insurance program with accreditation standards was associated with increases in achievement of minimal standards of prenatal care among women.	A	III-2	Pre- and post- evaluation, longitudinal study	+++
Lamb et al. (2003) [29]	United States	Acute care	To investigate how hospitals are dealing with the JCAHO standard that requires that all unanticipated outcomes of care are disclosed.	Survey (Completion rate 51%)	The vast majority of risk managers reported that their hospital’s practice was to disclose harm at least some of the time, although only one third of hospitals actually have board-approved policies in place. More than half of respondents reported they would always disclose death or serious injury, but when presented with actual clinical scenarios, respondents were much less likely to disclose preventable harms than to disclose non-preventable harms of comparable severity. Reluctance to disclose preventable harms was twice as likely to occur at hospitals having major concerns about malpractice implications of disclosure.	A	IV	Stratified random sample of hospitals	++
Longo et al. (1995) [32]	United States	Acute care	To examine compliance and characteristics of hospitals with tobacco control standards enacted by the Joint Commission on Accreditation of Healthcare Organisations. (JCAHO)	Onsite assessment of hospitals during period 1992–3 (N = 3327) Archival data	Two years after implementation, 95.6% of hospitals met the new JCAHO smoking ban standard; 90.9% of hospitals were in compliance with a second smoking standard requiring development and use of medical criteria for physician-ordered exceptions to the ban. Hospitals in tobacco-producing states had higher-than-average rates of compliance when compared to hospitals in other states. Hospitals providing psychiatric and/or substance abuse services had lower-than-average rates of compliance.	A	IV	Cross sectional	+++
					This first industry-wide smoking ban has been successful. However, hospitals should consider evaluating the use of medical exceptions to this policy.				
Piontek et al. (2003) [26]	United States	Acute care	To compare the impact of trauma patient outcomes before and after Level II American College of Surgeons (ACS) verification was received in a not-for-profit community hospital.	Database analysis	Study variable exhibited statistically different outcomes: length of stay (LOS) 10% less (p < 0.000); ratio of costs was 5% lower (p < 0.000); and mortality observed /expected ratios significantly different (0.81 before versus 0.59 after [p < 0.000]). The resources consumed achieving ACS Level II trauma centre verification resulted in decreased LOS, reduced in-hospital mortality rates, reduced cost and improved contribution margins.	A	III-3	Case control	++
Rowe-Murray and Fisher (2003) [20]	Australia	Acute care	To test the hypothesis that hospital practices in the immediate post partum period that are associated with operative intervention in delivery can affect the implementation of the Baby Friendly Hospital Initiative Step Four.	Prospective longitudinal study Interview (n = 203) Document analysis Survey	Women who had a caesarean section experienced significant delay in initiating breastfeeding compared with women giving birth vaginally with or without instrumental assistance (p < 0.001).	A	II	Prospective cohort	++
					Significant differences were observed among hospitals with Baby Friendly performing significantly better than the other 3 hospitals (p < 0.001). Birth delivery affected the implementation of Baby Friendly Hospital Initiative Step Four.				
Salmon et al. (2003) [27]	South Africa	Acute care	To test the impact of accreditation.	Survey (hospital organisational process indicator data)	Two years after the introduction of accreditation, the intervention group compliance with standards increased (38% to 76%) and the control group maintained level (37% top 38%). The accreditation program facilitated public hospitals’ compliance with standards.	I	II	Randomised control trial	++
Stradling et al. (2007) [34]	United States	Acute care	To examine stroke care delivery before and after Joint Commission stroke center certification.	Document analysis Database analysis	Certification improved clinical care (testing and medication) for patients with ischemic stroke. Clinical care improved with the certification of stroke centres.	A	IV	Pre- and post- evaluation	++
Thornlow and Merwin (2009) [18]	United States	Acute care	To examine the relationship between patient safety practices, as measured by accreditation standards, and patient safety outcomes as measured by hospital rates of infections, decubitus ulcers, postoperative respiratory failure, and failure to rescue.	Database analysis (secondary data)	Accreditation standards reflecting patient safety practices were related to some outcomes, but not others. Rates of infections and decubitus ulcers occurred more frequently in hospitals with poorer performance in utilizing patient safety practices, but no differences were noted in rates of postoperative respiratory failure or failure to rescue. Certain adverse events, such as infections and decubiti, may be reduced by preventive protocols that are reflected in accreditation standards, whereas other events, such as failure to rescues and postoperative respiratory failure, may require multifaceted strategies that are less easily translated unto protocols.	A	IV	Cross-sectional	+++
Valenstein et al. (2009) [31]	United States	Acute care	To determine how document control is being implemented in practice and whether particular approaches result in better levels of compliance.	Document analysis	35% fulfilled all 6 document control requirements (3113/8814); 97% met the requirements for the availability of the document; 50% fulfilled archiving requirements. Policies and procedures were more likely to fulfil document control requirements than forms and work aids. Document control practices significantly associated with higher compliance rates were unable to be identified. Most laboratories are not meeting regulatory and accreditation requirements related to the control of documents.	A	IV	Prospective cross sectional	++
Weng et al. (2003) [28]	Taiwan	Acute care	To examine the effect of the Baby Friendly Hospital Initiative (BFHI) on the Taiwanese breastfeeding rate and analyse factors related to BFHI qualification.	Document analysis	Mothers in qualified BFHI had higher breastfeeding rates than those in non-qualified hospitals whether they were surveyed while in maternity wards after delivery (88.1% vs 78.1%) or in their first post-natal month (67.6% vs 59.4%). Close correlation between BFHI qualification and location and grade of hospital. Factors related to qualification were: hospital fosters establishment of breastfeeding support groups; written breastfeeding policy; practise rooming-in available 24 hours a day; and health staff trained. Health policy intervention has had a significant impact on increasing the breastfeeding rate in Taiwan.	A	IV	Retrospective cross sectional	++

### Study details, characteristics and quality

The dates of the studies ranged from 1995 to 2009 inclusive. The majority of studies were published in two years, 2003 [20,26-29] and 2009 [17,18,30,31], with five and four studies, respectively. One study was published in each of the following years: 1995 [32], 2004 [33], 2007 [34] and 2008 [35]. Studies were conducted in six countries. The United States of America (USA) was the setting for the majority of studies (n = 8) [17,18,26,29,31-34]. The remaining five countries all had one study: United Kingdom [35]; Philippines [30]; Australia [20]; South Africa [27]; and Taiwan [28]. The studies were all conducted in the acute sector (n = 13). The majority of studies had a multidisciplinary focus (n = 9) [17,18,20,26-28,32-34] and the practices of nurses [30,35] and managers [29,31] were the individual focus of two studies each. Research projects used mixed methods [20,32-35], employed quantitative methodologies to examine archival databases [17,18,26,28,31] or undertook a questionnaire survey [27,29,30]. Within the mixed methods studies the qualitative tools were questionnaires, surveys, interviews, reviews and evaluations. The quantitative methods covered examination of databases, prospective and retrospective studies and stratified randomised studies. The study content was categorised according to the focus of the papers, that is, program, clinical or workplace issues. Program issues was the topic that most studies examined via four different program sub-topics: reviews of programs (n = 5) [18,20,28,29,31]; policy compliance (n = 4) [17,32-34]; program impacts (n = 3) [26,27,30]; and organisational environment (n = 1) [35]. Just five studies had content relating to clinical care [17,18,20,26,34] and one on staff workplace issues [35].

A summary of the intervention or impact (aetiology) assessment, level of evidence classification and quality ratings for the selected literature is represented in Table 3. Using the NHMRC guidelines, three investigations [27,32,35] were classified as interventions and ten studies [20,26,28-33,36] under the aetiology criteria. In the intervention group, Aiken et al. (2008), was assessed as meeting the fourth level of evidence and all the quality criteria. While Salmon et al. (2003) and Stradling et al. (2007) were rated at the second and fourth level of evidence rating, respectively, each were missing some study details and so were rated at the second level for quality ratings. The studies within the aetiology group were divided between the two top quality levels. Six [26,29,30,32,33,36] were rated as meeting all criteria, and four [28,29,31,37], while missing some but not significant information to compromise them, were rated on the second tier of quality. 

**Table 3:** Summary of the intervention or aetiology assessment, level of evidence classification and quality ratings

**Level of evidence**		**Quality ratings**	
	+++	++	+
**I**			
**II**		**Salmon et al. (2003)**[27]	
		Rowe-Murray and Fisher (2003) [20]	
**III-2**	Herr and Titler (2009) [17] Kozhimann et al. (2009) [30]		
**III-3**	Piontek et al. (2003) [26]		
**IV**	**Aiken et al. (2008)**[35]	**Stradling et al. (2007)**[34]	
	Devers et al. (2004) [34]	Lamb et al. (2003) [29]	
	Longo et al. (1995) [32]	Valenstein et al. (2009) [31]	
	Thornlow and Merwin (2009) [18]	Weng et al. (2003) [28]	

**Bold = Interventions as per the NHMRC criteria;** Non-bold = Aetiology as per the NHMRC criteria.

### Standards development

No study directly examined standards development or other issues associated with their progression. That is, no empirical study was identified which examined: what is best practice for developing standards; standard development processes; the wording or structure of standards; or what types of standards would have the greatest likelihood of improving practice.

### Implementation issues

Only one study examined implementation issues with healthcare accreditation standards [33]. Five factors were noted as assisting implementation: external pressure from legislation and accreditation; the use of technology and self-evaluation as tools to leverage change; organisational culture characteristics; research; and peer education. Conversely, three factors were reported to hinder implementation: lack of external incentives or pressure; organisational policies and culture; and cost and resource constraints [33].

### Impact of standards

Twelve of the 13 papers addressed the impact of standards [26-32,35-37]. The impact of the standards on the organisation, clinical quality and staff could be identified.

#### Impacts of standards on the organisation

The single randomised controlled trial identified demonstrated that compliance with accreditation standards increased in the intervention group, from 38 to 76%, compared to in the control group, from 37 to 38% [27]. Furthermore, standards or guidelines about the organisation of clinical practice led to improved efficiency and quality practices. Specifically, standards within an accreditation program resulted in decreased length of hospital stay [26], improved management of disclosure of preventable harm [29], and utilisation of patient safety practices [36].

#### Impacts of standards on clinical quality

Accreditation program standards encompassing trauma care [26], prenatal care [30], post partum care [37], stroke care [32], breastfeeding [28], pain management [29], and the institution wide organisation of care [27,30,33] were reported to improve the provision of care. Additionally, there were links to improvements in various aspects of clinical quality. For example, standards contributed to: reductions in in-hospital mortality and length of stay [26], and rates of infections and decubitus ulcers [36]; and improvements in breastfeeding rates [28] and the proportion of patients receiving relevant tests, medications and admission for stroke [32]. Conversely, and at times simultaneously, standards introduced to improve care appeared not to do so. For example, exposure to standards for prenatal and delivery care [30], document control [31], and the organisation of care [27] did not show any measurable effects. Nor did rates of certain adverse events, such as failure to rescue or postoperative respiratory failure, alter with the implementation of accreditation standards [36].

#### Impact on staff

Standards were shown to produce an improved staff quality of life, working conditions and appraisals of the quality of care. This outcome was noted from the use of ‘Magnet’ principles, which sought to improve the attraction of the workplace in recruiting and retaining staff [35]. Additionally, the introduction of standards, through an accreditation program, resulted in the improved perceptions of teamwork and participation in decision making [27], and compliance with tobacco control [32].

## Discussion

This study employed systematic search procedures to academic databases and accreditation agency websites to uncover empirical research that grounds the development methods and application of healthcare accreditation standards. The review has built on previous work in the healthcare accreditation field [16,17], commencing where previous reviews finished. We started with a proposition that standards are ubiquitous within healthcare and are generally considered to be an important means by which to improve clinical practice and organisational performance. However, the evidence about whether accreditation standards change behaviour of health care organisations, clinical quality and staff is at best equivocal, and is determined by the circumstances.

Only three intervention studies were identified in the review. Two interventions resulted in improvements attributed to the implementation of accreditation standards [32,35]. The improvements were the organisational working environment and staff perceptions [35], and care processes and appropriateness of care [32]. The remaining study, conducted in a developing country [27], involved health services seeking improvement from a very low base and hence the applicability of the results is limited to that context. The non-intervention studies have shown that, whilst there is adherence to standards in some cases, in a range of instances there is little evidence as to their effects. In short, the effectiveness of the development, writing, implementation and impacts of healthcare standards are significant issues that lack convincing evidence.

It is not clear, for example, what might be evidence-based practice in the development of standards. However, the literature synthesis suggests that reoccurring strategies include mobilising external leverage, organising teams or creating receptive cultures within health care organisations to optimise the opportunity to create standards. Yet an overarching finding was that applying standards has mixed results. There is limited published peer-reviewed evidence regarding the correspondence between the application of standards and improvements in organisational performance, clinical quality or staff behaviours.

There is the opportunity for the standards development field to learn from the experience of people developing technical standards, practice guidelines and evidence-based clinical policies. Consideration can be given to the applicability of translation of development processes and implementation strategies from other areas in healthcare [38-40]. The Joint Commission in the USA, for example, through the establishment of the National Patient Safety Goals initiative has used development and implementation processes from which lessons can be learnt [41].

Agencies setting standards, including accreditation bodies or programs that develop or apply them, or both, also have significant experience and expertise in conducting these activities. Some have been doing so for decades. More recently, ISQua is utilising and sharing this experience through two strategies: the ISQua IAP and the accreditation workshop conducted at ISQua’s annual international quality conference. The ISQua IAP has been implemented to “build credibility and comparability for national organisations by harmonising standards and procedures on common international principles” [42]:349. Established in 1999, the IAP utilises the expertise of senior people within accreditation agencies to review, offer ideas for improvements, and accredit programs in other countries. ISQua reports that the IAP has accredited 19 organisations and 35 sets of standards (from 21 organisations), and eight surveyor training programs [36]. Each year the accreditation workshop at the ISQua international quality conference draws together practitioners and researchers from around the world to consider current developments and challenges associated with healthcare accreditation programs [43]. Discussions have centred upon all aspects of accreditation programs, for example: implementation of accreditation programs [44,45]; maintaining standards of accreditation programs [46]; survey methodologies [47,48]; linking standards to clinical indicators [49]; processes used to develop standards [50]; and the public disclose of accreditation results [51,52].

## Conclusion

The challenge is to translate practical experiences and discussions into rigorous empirical evidence. We lack knowledge of how to strengthen the development of standards and the application of them based on sound critically peer-reviewed evidence. The process to develop standards essentially needs to be transformed from learnt experience to a verifiable, evidence-based methodology. Evidence-based mechanisms by which standards are developed, promulgated, reinforced, audited and evaluated are needed. Linking the writing of standards, including the wording, structure, design, focus and content, to improved outcomes requires further rigorous investigation. Factors that promote or inhibit implementation of standards, and the impacts that result, need detailed examination and analysis. This review has revealed some significant gaps in our knowledge in these areas, and, in doing so, extended previous reviews in the healthcare accreditation field.

As to the limitations of our study, while we have endeavoured to be systematic, we may have overlooked some important literature. A further limitation is that papers or reports needed to be publicly available and in English to be included in the results.

## Competing interests

The authors declare that they have no competing interests.

## Authors’ contributions

DG and MP performed the literature search, and along with RH selected relevant papers for the review and analysed the included papers. DG, MP and RH drafted the initial manuscript, and all authors contributed to the revision of the manuscript. All authors read and approved the final manuscript.

## Pre-publication history

The pre-publication history for this paper can be accessed here:

http://www.biomedcentral.com/1472-6963/12/329/prepub

## Supplementary Material

Additional file 1 ** Appendix 1. **Accreditation and Standards Agencies websites searched. Click here for file
