# Supplementary material for: The standard of healthcare accreditation standards: a review of empirical research underpinning their development and impact
Source: BMC Health Serv Res. 2012 Sep 20;12:329. doi: 10.1186/1472-6963-12-329 (PMC3520756; doi:10.1186/1472-6963-12-329)
Supplement: Additional file 1 — Appendix 1. Accreditation and Standards Agencies websites searched. [file 1472-6963-12-329-S1.doc]

**Additional file 1** Appendix 1

**Accreditation and Standards Agencies websites searched**

| **Accreditation Agencies** | **Websites** |
| --- | --- |
| Accreditation Canada | www.accreditation.ca |
| Aged Care Standards and Accreditation Agency, Australia (ACSAA) | www.accreditation.org.au |
| American Association of Blood Banks, Accreditation and Quality Programme (AABB) | www.aabb.org |
| Australian Council on Healthcare Standards (ACHS) | www.achs.org.au |
| Australian General Practice Accreditation Limited (AGPAL) | www.agpal.com.au |
| Centrum Monitorowania Jakosci w Ochronie Zdrowia (Poland) | www.cmj.org.pl |
| Commission on Accreditation of Rehabilitation Facilities (CARF) (United States) | www.carf.org |
| Community Health Accreditation Body (Canada) | Not accessible |
| Consórcio Brasileiro de Acreditação (CBA) (Brazil) | www.cbacred.org.br |
| Council for Health Service Accreditation of Southern Africa (COHSASA) | www.cohsasa.co.za |
| Fundación Avedis Donabedian (FAD) (Spain) | www.fadq.org |
| Healthcare Accreditation Quality Unit, UK (CHKS-HAQU) | www.chks.co.uk |
| Health Accreditation System of Instituto Colombiano de Normas Técnicas y Certificación, Co (ICONTEC) | www.icontec.org.co |
| Health and Disability Auditing New Zealand (HDANZ) | www.healthaudit.co.nz |
| Haute Autorité de santé (HAS) | www.has-sante.fr |
| Health Care Accreditation Council of Jordan (HCAC) | www.hcac.jo |
| International Society for Quality in Health Care (ISQua) | www.isqua.org |
| Instituto da Qualidade em Saude (IQS) (Portugal) | www.spqsaude.com |
| Irish Health Service Accreditation Board (IHSAB) | Not accessible |
| Italian Society for Quality of Health Care (ITAES) | Not accessible |
| Japan Council for Quality Health Care | www.jcqhc.or.jp |
| Joint Commission International (JCI) | www.jointcommissioninternational.org |
| Joint Commission (TJC) (United States) | www.jointcommission.org |
| KTQ (Kooperation für Transparenz und Qualität im Gesundheitswesen GmbH) (Germany) | www.ktq.de |
| Malaysian Society for Quality in Health (MSQH) | www.msqh.com.my |
| National Accreditation Board for Hospitals & Healthcare Providers (NABH) (India) | www.nabh.co |
| Netherlands Institute for Accreditation in Healthcare (NIAZ) | www.en.niaz.nl |
| Quality Improvement Council and the QIC Accreditation Program (QIC) | www.qic.org.au |
| Taiwan Joint Commission on Healthcare Accreditation (TJCHA) | www.tjcha.org.tw |
| Technical Institute for Accreditation of Healthcare Organisations (Argentina) | Not accessible |
| Telarc Quality Health New Zealand | www.telarcqualityhealth.co.nz |
| **Standard Agencies** |  |
| International Standards Organisation (ISO) | www.iso.org |
| American National Standards Institute | www.ansi.org |
| Standards Australia | www.standards.org.au |
| Accreditation Board for Standards Development Organisations | www.absdo.org.au |
| Global-Mark Pty Ltd | www.global-mark.com.au |
